# Characterization of stem cell landscape and identification of stemness-relevant prognostic gene signature to aid immunotherapy in colorectal cancer

**DOI:** 10.1186/s13287-022-02913-0

**Published:** 2022-06-09

**Authors:** Hang Zheng, Heshu Liu, Huayu Li, Weidong Dou, Jingui Wang, Junling Zhang, Tao Liu, Yingchao Wu, Yucun Liu, Xin Wang

**Affiliations:** 1 Department of General Surgery, Peking University First Hospital, Peking University, Beijing, People’s Republic of China; 2 Department of Oncology, Beijing Chaoyang Hospital, Capital Medical University, Beijing, People’s Republic of China

**Keywords:** Colorectal cancer, Stemness, Bioinformatics, Tumor microenvironment, Immunotherapy

## Abstract

**Background:**

It is generally accepted that colorectal cancer (CRC) originates from cancer stem cells (CSCs), which are responsible for CRC progression, metastasis and therapy resistance. The high heterogeneity of CSCs has precluded clinical application of CSC-targeting therapy. Here, we aimed to characterize the stemness landscapes and screen for certain patients more responsive to immunotherapy.

**Methods:**

Twenty-six stem cell gene sets were acquired from StemChecker database. Consensus clustering algorithm was applied for stemness subtypes identification on 1,467 CRC samples from TCGA and GEO databases. The differences in prognosis, tumor microenvironment (TME) components, therapy responses were evaluated among subtypes. Then, the stemness-risk model was constructed by weighted gene correlation network analysis (WGCNA), Cox regression and random survival forest analyses, and the most important marker was experimentally verified.

**Results:**

Based on single-sample gene set enrichment analysis (ssGSEA) enrichments scores, CRC patients were classified into three subtypes (C1, C2 and C3). C3 subtype exhibited the worst prognosis, highest macrophages M0 and M2 infiltrations, immune and stromal scores, and minimum sensitivity to immunotherapies, but was more sensitive to drugs like Bosutinib, Docetaxel, Elesclomol, Gefitinib, Lenalidomide, Methotrexate and Sunitinib. The turquoise module was identified by WGCNA that it was most positively correlated with C3 but most negatively with C2, and five hub genes in turquoise module were identified for stemness model construction. CRC patients with higher stemness scores exhibited worse prognosis, more immunosuppressive components in TME and lower immunotherapeutic responses. Additionally, the model’s immunotherapeutic prediction efficacy was further confirmed from two immunotherapy cohorts (anti-PD-L1 in IMvigor210 cohort and anti-PD-1 in GSE78220 cohort). Mechanistically, Gene Set Enrichment Analysis (GSEA) results revealed high stemness score group was enriched in interferon gamma response, interferon alpha response, P53 pathway, coagulation, apoptosis, KRAS signaling upregulation, complement, epithelial–mesenchymal transition (EMT) and IL6-mediated JAK-STAT signaling gene sets.

**Conclusions:**

Our study characterized three stemness-related subtypes with distinct prognosis and TME patterns in CRC patients, and a 5-gene stemness-risk model was constructed by comprehensive bioinformatic analyses. We suggest our stemness model has prospective clinical implications for prognosis evaluation and might facilitate physicians selecting prospective responders for preferential use of current immune checkpoint inhibitors.

**Supplementary Information:**

The online version contains supplementary material available at 10.1186/s13287-022-02913-0.

## Introduction

The morbidity and mortality of colorectal cancer (CRC) patients are both estimated to rank third worldwide, which pose a grave threat to human health [1]. Although CRC might be cured by radical surgery combined with chemo- and radiotherapy, drug resistance, recurrence and metastasis are still the main causes of CRC-associated mortality after R0 resection. Mounting evidence has demonstrated that CRC originates from cancer stem cells (CSCs) [2–4], which account for a small subpopulation of tumor mixture and are characterized by self-renewal, unrestrictive proliferation, multidirectional differentiation and tumorigenesis initiation [2, 5, 6]. CSCs are capable of forming disseminated metastatic tumors owing to their expansive proliferative capability [7], and it is well acknowledged that CSCs are principally responsible for CRC progression, metastasis and therapy resistance [8, 9], which make them as promising therapeutic targets [10].

While several CSCs biomarkers have been identified for CRC [11, 12], unfortunately, CSCs are functionally and phenotypically heterogeneous populations that extending reversibly from pluripotent to differentiated cells and existing both between patients and within a single tumor [13–15], which have precluded clinical application of CSC-targeting therapy. In addition, tumor microenvironment (TME) could interactively influence CSCs through intricate intercellular cross-talking, and CSCs are characterized by low immunogenicity and suppressed immune response in CRC [16–18]. Studies have reported that more TGF-β was secreted by breast [19] and glioblastoma [20] CSCs as compared to regular tumor cells. Colon cancer CSCs could produce IL-4 to induce anti-apoptosis of CSCs and undermine CD8+ T cell-mediated antitumor immune response [21, 22]. Under inflammatory conditions, immune cells would also release various inflammatory cytokines like IL-1, IL-4, IL-6, IL-8, IL-10 and TGF-β [23], which generate a positive feedback inflammatory loop through the bilateral activations of Stat3/NF-κB pathways to sustain a chronic inflammatory state and stimulate the self-renewal of CSCs [24]. Therefore, a comprehensive depiction of the highly heterogeneous CSCs and their adaptable and dynamic cross-talks with the TME landscape would aid in the exploration of CSC-targeted therapeutic strategies as well as sensitization of current immunotherapies.

In this study, based on public 26 stemness gene sets, single-sample gene set enrichment analysis (ssGSEA) algorithm was applied to portray an expansive outlook about the stemness landscape from gene expression profiles of bulk CRC samples, and three stemness subtypes with discrete stemness and TME features were identified by unsupervised clustering method. Subsequently, genes highly correlated with stemness subtypes and prognosis were identified by weighted gene correlation network analysis (WGCNA). The stemness-risk model was then constructed based on Cox regression and random survival forest analyses, and its correlations with prognosis, TME patterns, molecular functions and chemotherapy/immunotherapy efficacies in CRC were further investigated. Conclusively, we established the stemness-risk score to characterize the stemness landscapes, which could robustly predict prognosis and response to immunotherapy for CRC patients.

## Materials and methods

### CRC datasets acquisition and pre-processing

A total of 1,467 CRC samples datasets with corresponding clinical and survival annotations were procured form five cohorts: TCGA-COAD, TCGA-READ, GSE39582, GSE17536 and GSE103479. The gene expression data (fragments per kilobase per million mapped reads (FPKM) standardized data) of TCGA-COAD and TCGA-READ were downloaded from the GDC hub of UCSC Xena browser (https://gdc.xenahubs.net) [25] and transformed to transcripts per million (TPM) values, as TPM modifies the inconsistency of gene lengths and qualifies for comparisons among samples [26]. The other three RNA‐Seq datasets (GSE39582 [27], GSE17536 [28] and GSE103479 [29]) were obtained from the Gene Expression Omnibus (GEO, http://www.ncbi.nlm.nih.gov/geo/) database and preprocessed through Robust Multichip Average algorithm [30]. The Combat function of sva R package (v3.35.2) was implemented to eliminate batch effects in these high-throughput experiments [31].

### Stemness signatures collection and consensus clustering for CRC stemness subtypes

Firstly, we recruited 26 stemness gene sets from a web-based tool: StemChecker (http://stemchecker.sysbiolab.eu/) [32], which was based on the most comprehensive and updated collection of published stemness signatures defined by gene expression profiles, RNAi screens, transcription factor (TF) target gene sets, literature and computational summaries (Additional file 2: Supplementary Table 1). Then, ssGSEA was implemented to quantitatively elucidate the stemness enrichment scores of the 26 stemness gene sets in each CRC sample via GSVA R package (v1.34.0) [33]. Subsequently, based on each sample’s ssGSEA scores, we performed consensus clustering algorithm for unsupervised classification of CRC samples through ConsensusClusterPlus R package (v1.50.0) [34]. K-means (km) cluster method upon Euclidean distance was applied in this analysis and was repeated for 1000 iterations to ensure dependability.

### Tumor microenvironment (TME) infiltrations exploration

CIBERSORT deconvolution algorithm could robustly quantify the relative proportions of 22 immune cells from normalized bulk sample’s gene expression profiles [35]. We quantified the TME fractions of each CRC sample via “CIBERSORT” R script with the LM22 leukocyte gene signature and 1,000 permutations. Samples with CIBERSORT P value less than 0.05 were screened. In addition, we also used ESTIMATE algorithm to calculate immune and stromal scores of each CRC sample via estimate R package (v1.0.13), which were representative of the immune and stromal cellular infiltrations in each sample [36].

### Chemotherapy sensitivity and immunotherapy response predictions

The half-maximum inhibitory concentration (IC50) values of several drugs (Bleomycin, Bosutinib, Camptothecin, Cisplatin, Cytarabine, Docetaxel, Elesclomol, Etoposide, Gefitinib, Gemcitabine, Lapatinib, Lenalidomide, Methotrexate, Paclitaxel, Sunitinib) in each CRC sample were computed for the prediction of chemical sensitivity via pRRophetic R package (v0.5), which was based on Genomics of Drug Sensitivity in Cancer (GDSC) (https://www.cancerrxgene.org/) by using ridge regression [37, 38]. And tenfold cross-validation was applied for the prediction accuracy evaluations. In addition, Tumor Immune Dysfunction and Exclusion (TIDE), a reliable online algorithm (http://tide.dfci.harvard.edu/) qualified for immunotherapeutic response predictions, was employed to estimate immunotherapeutic responses of each CRC patient [39]. Moreover, we also downloaded and analyzed two real-world immunotherapeutic cohorts: the IMvigor210 dataset from http://research-pub.gene.com/IMvigor210CoreBiologies, which contains the microarray, survival and anti-PD-L1 immunotherapy data of metastatic urothelial cancer patients [40], and GSE78220 cohort contains transcriptional data of metastatic melanoma patients treated with anti-PD-1 therapy [41].

### WGCNA and candidate hub genes identification

The WGCNA R package (v1.68) was employed to identify co-expressed gene networks that were representative of diverse stem cell subtypes in GSE39582 dataset [42]. The median absolute deviation (MAD) top 5000 genes were screened for network constructions, and co-expression similarity matrix (*s*_ij_) was computed by the Pearson’s correlation coefficient between any two genes (*x*_i_ and *x*_j_):

$${s}_{ij}=|\mathrm{cor}\left({x}_{i},{x}_{j}\right)|$$

Then, a weighted adjacency matrix (*a*_ij_) was calculated by raising *s*_ij_ to a soft thresholding power *β* = 7 (Fig. 4a):

$${a}_{ij}={{s}_{ij}}^{\beta }$$

Subsequently, a topological overlap matrix (TOM) and correlative dissimilarity matrix (1-TOM) were built from the adjacency matrix to cluster highly interconnected genes into various gene modules (minModuleSize was set as 30) [43]. Later, module eigengene (ME) was calculated, which represented the first principal component of each module, and the associations of modules with each stemness subtype, TNM stage and survival status were determined. Parameters of hub genes of the specific module were set as gene significance (GS, Pearson’s correlation between each gene and clinical trait) > 0.4 and module membership (MM, correlation between each gene and module) > 0.8. Afterward, Gene Ontology (GO) and Kyoto Encyclopedia of Genes and Genomes (KEGG) analyses of hub genes in the co-expression module were performed by clusterProfiler R package (v3.14.3) [44].

### Prognostic stemness model construction and validation

Univariate Cox regression analysis was employed to screen overall survival (OS)-associated hub genes (*P* < 0.05) of the interested module in GSE39582 dataset. Afterward, we created random survival forest (RSF) model for model reduction by using rfsrc function of randomForestSRC R package (v2.9.3). We preferred this model in that random forest could robustly quantify the relative significance of each variable, and genes with relative importance > 0.5 were incorporated. Next, a gene combination with the optimal log-rank *P* value was identified for the signature construction: stemness-risk score = Ʃ (*βi* * Expi), where *βi* was the ith gene’s Cox coefficient, and Expi was the ith gene’s expression value. The prognostic value of the stemness-risk score was then validated in TCGA-COAD, TCGA-READ, GSE17536, GSE103479, the entire CRC patients and IMvigor210 cohort patients.

### Messenger RNA expression-based stemness index (mRNAsi) calculation

The transcriptional mRNAsi of each CRC sample (ranges from zero to one) was computed following the method of Malta et al. using one-class logistic regression machine-learning algorithm (OCLR) based on pluripotent stem cell samples, which strongly correlated with stem cell features and could be applied for cancer stemness predictions [45]. The prognostic value of mRNAsi indices as well as the Spearman correlation between stemness-risk score and mRNAsi indices was analyzed in all 1,467 CRC patients.

### Hallmark gene sets enrichment analyses

The total 1,467 CRC patients were separated into high and low stemness-risk groups according to the median stemness-risk score. Gene set enrichment analysis (GSEA) was performed to explore the functionally enriched pathways and hallmark gene sets related to subgroups via clusterProfiler R package [44] [46], and the hallmark (h.all.v7.3) gene sets were downloaded from the Molecular Signatures Database (MSigDB, http://software.broadinstitute.org/gsea/msigdb/). *P* < 0.05 was considered as significantly enriched.

### Cell culture and siRNA transfection

Human CRC cell lines SW480, SW620, Caco-2 and HCT116 were purchased from the Cancer Institute of the Chinese Academy of Medical Sciences. SW480, SW620 and Caco-2 cells were cultured in Dulbecco’s modified Eagle’s medium (DMEM, Biological Industries, Israel). HCT116 was cultured in McCoy’s 5A Medium (Biological Industries, Israel). All cells were supplemented with 10% fetal bovine serum (FBS, Biological Industries, Israel) and 1% penicillin–streptomycin (Biological Industries, Israel) and cultured in 5%CO_2_ incubator at 37 °C. Small interfering RNA (siRNA) against COLEC12 (si-COLEC12) and the corresponding negative control (NC) were synthesized by GenePharma (Jiangsu, China), and sequences of siRNA were as follows: si-COLEC12-1 (sense 5′–3′): GCAAUCUGCAGAACCAAAUTT; si-COLEC12-2 (sense 5′–3′): GCGAAUCAAGAACGACUUUTT; si-COLEC12-3 (sense 5′–3′): GCUGACCAGCAAUCUAAAUTT; si-NC (sense 5′–3′):UUCUCCGAACGUGUCACGUTT. Following the manufacturer’s instructions, CRC cells cultured in 6-well plates were transfected at 70%–80% confluence using Lipofectamine 3000 (Invitrogen, USA), and the knockdown efficiency of COLEC12 protein expression was measured with goat anti-human COLEC12 polyclonal antibody (R&D Systems, USA) by western blotting (WB) after 48 h of cultivation.

### Western blotting

Detailed WB protocol was previously described [47]. Briefly, CRC cell lines were lysed in RIPA lysis buffer. BCA assay kit (Beyotime, Shanghai, China) was utilized for measuring protein concentration, and 30 μg of total protein was separated by 10% SDS-PAGE and electroblotted onto PVDF membranes (Millipore, Burlington, MA, USA). The membranes were then blocked with 5% milk for 1 h at room temperature and incubated with primary antibody at 4 °C overnight, followed by incubated with secondary antibodies (1:8000, ZSGB-BIO, Beijing, China) for 1 h at room temperature. The following primary antibodies were utilized: anti-SOX2 (1:1000, Cell Signaling Technology), anti-LGR5 (ab273092, 1:1000, Abcam), anti-OCT4 (C52G3, 1:1000, Cell Signaling Technology), anti-Nanog (D73G4, 1:1000, Cell Signaling Technology), anti-CD44 (156-3C11, 1:1000, Cell Signaling Technology), anti-GAPDH (abs830030, 1:1000, UNIV).

### Tumorsphere formation assay

Caco-2 cells (5 × 10^3^ cells/well) were seeded into an ultralow-attachment six-well plate with 1.5 ml of sphere-culturing medium containing DMEM-F12 medium (Gibco, Milan, Italy) with penicillin/streptomycin, L-glutamine (2 Mm, #7100, Stem cell), Insulin (5ug/ml, # P3376-100 IU, Beyotime), BSA (4 mg/ml, #ST025-5 g, Beyotime), Glucose (6 mg/ml), bFGF (10 ng/mL, PHG0024, Gibco) and Recombinant Human EGF (20 ng/mL, PHG0311, Gibco). Tumorspheres were observed and photographed under microscope after 3–5 days of culture.

### Statistical analyses

All statistical analyses were performed in R software (v3.6.3). Wilcoxon test was used for pairwise comparison between two groups, and Kruskal–Wallis test was used for multiple groups comparisons. Kaplan–Meier method and log-rank test were performed for survival analysis. The optimal cutoff value of the stemness-risk score was determined by the “surv_cutpoint” function of survminer R package (v0.4.6). A *P* value < 0.05 was regarded as statistically significant.

## Results

### Landscape of stem cell gene sets enrichments and identification of three stemness subtypes with different survival outcomes and immune infiltrations

**Fig. 1:**
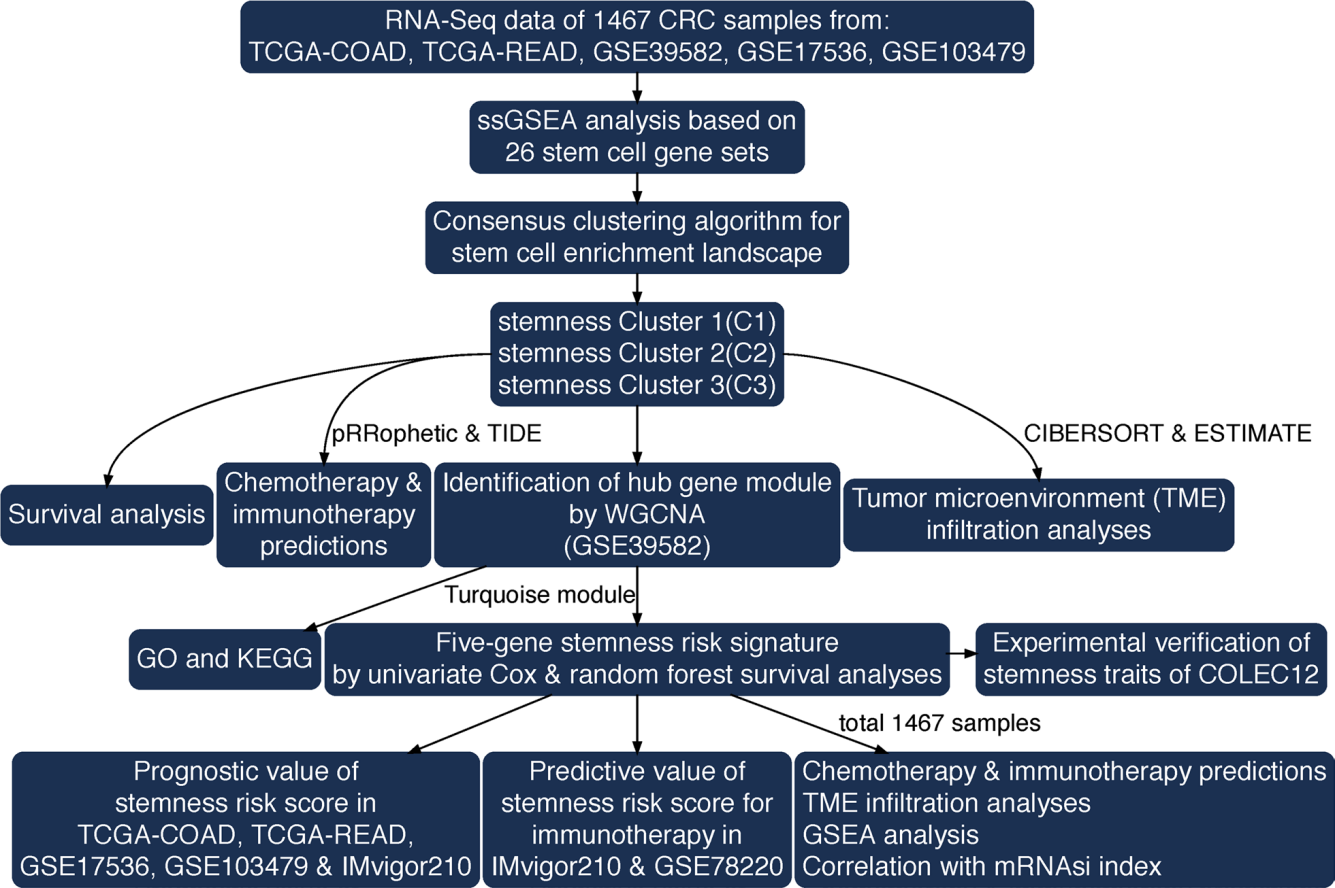
Work flow of this study

**Fig. 2:**
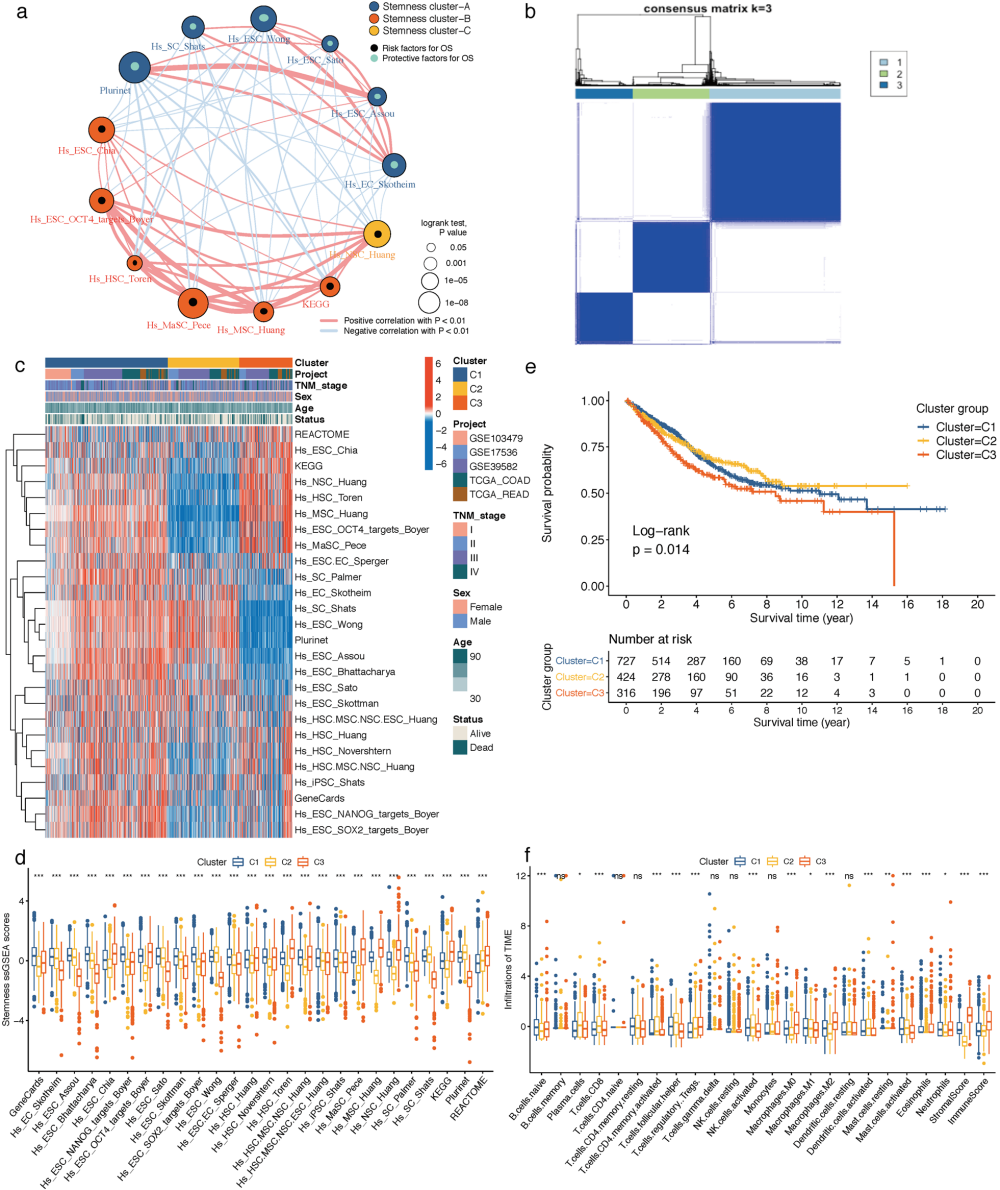
**a** Prognostic stemness network landscape of 13 prognostic stemness gene sets interactions, lineages and their impacts on OS for CRC patients. The size of each gene set represents its prognostic impact by log-rank test. Skyblue dots indicate favorable variables and black dots represent risk factors. The red lines connecting stemness gene sets indicate positive Spearman correlations, and the blue lines are negative correlations, and the thickness of the lines represents the Spearman correlation strengths. **b** Consensus clustering identified three distinct clusters of CRC with different stemness gene sets enrichment scores. **c** Heatmap depicting the landscape of ssGSEA stemness scores in three clusters. **d** Box plot displaying the differences of 26 ssGSEA stemness scores between the three clusters by Kruskal–Wallis test.  ***P < 0.001. **e** Kaplan–Meier OS curves for CRC patients between different stemness subtypes. **f** Box plot showing the differences of 22 infiltrating immune cells, stromal and immune scores between the three clusters by Kruskal–Wallis test. ns, not significance, ∗*P* < 0.05, ∗∗*P* < 0.01,  ***P < 0.001. OS, overall survival; CRC, colorectal cancer

The overall construction scheme of stemness-enrichment patterns and stemness-risk signatures is displayed in Fig. 1. The enrichment scores of 26 stemness gene sets in each CRC sample were quantified by ssGSEA algorithm, and 13 prognostic stemness gene sets were firstly identified by univariate Cox analysis (*P* < 0.05) for the depiction of prognostic stemness network, which exhibited the landscape of prognostic stemness gene sets interactions, lineages and their impacts on OS for CRC patients (Fig. 2a). Next, based on the total 26 stemness gene sets ssGSEA scores, unsupervised clustering was conducted through ConsensusClusterPlus package to categorize CRC patients into three distinct clusters (C1-3, Fig. 2b). The Spearman correlations of the 26 stemness ssGSEA scores are shown in Additional file 1: Supplementary Fig. 1. Among the three stemness clusters, Cluster 1 (727 patients) had the widespread enrichment degree for most of the stemness gene sets; Cluster 2 (424 patients) exhibited high enrichments of stemness gene sets with favorable prognosis like Hs_ESC_Wong, Hs_SC_Shats and Plurinet; and Cluster 3 (316 patients) was significantly enriched in the adverse prognostic stemness gene sets like Hs_ESC_Chia, Hs_HSC_Toren, Hs_MaSC_Pece, Hs_MSC_Huang and Hs_NSC_Huang (Fig. 2c, d). Consistently, Kaplan–Meier analysis revealed that Cluster 3 CRC patients experienced worse OS compared with the other two clusters (log-rank *P* = 0.014, Fig. 2e).

To further elucidate the TME landscape among stemness subtypes, we performed CIBERSORT and ESTIMATE analyses to compare the TME fractions as well as immune and stromal scores. A total of 893 qualified CRC samples with CIBERSORT *P* < 0.05 were incorporated into the subsequent analysis. As shown in Fig. 2f, among the three stemness clusters, Cluster 3 exhibited an immunosuppressive subtype that was characterized by abundances of macrophages M0 and macrophages M2, as well as higher immune and stromal scores; Cluster 2 displayed higher anti-tumor TME components like CD8+ T cells, activated memory CD4+ T cells, T cells follicular helper, activated NK cells and activated dendritic cells; and Cluster 1 showed moderate TME infiltrations like activated memory CD4+ T cells, T cells follicular helper, activated NK cells, macrophages M0, macrophages M2, activated dendritic cells and stromal score.

### Chemotherapy sensitivity and immunotherapy response among stemness subtypes

**Fig. 3:**
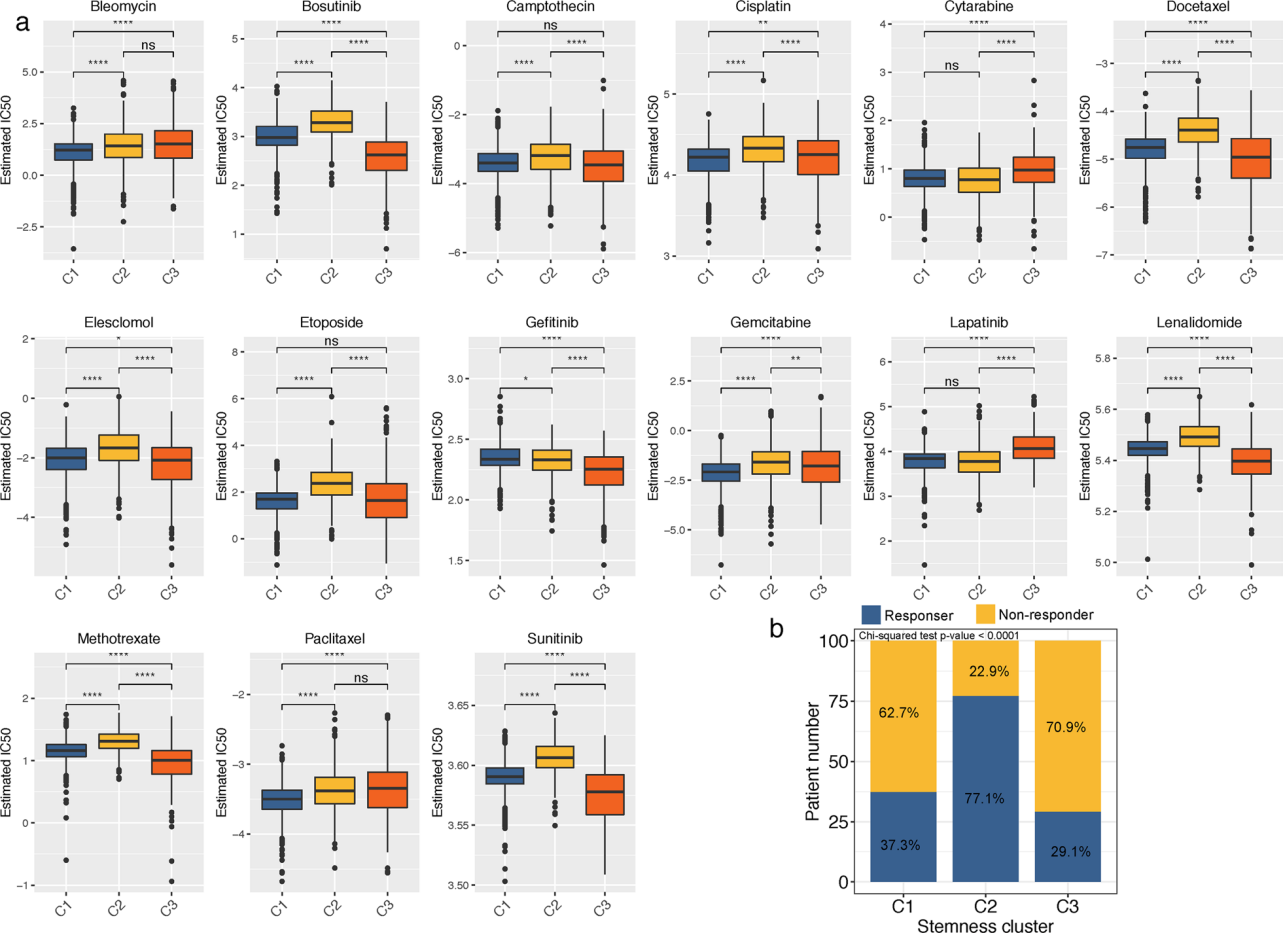
**a** Box plots for the estimated IC50 of chemotherapy drugs between three stemness subtypes. **b** Distributions of responder and non-responder to immunotherapy among distinct stemness clusters estimated by TIDE algorithm

Currently, surgery and systemic chemotherapy remains the conventional strategy for CRC patients. Therefore, we estimated IC50 values via pRRophetic algorithm for chemotherapy sensitivity evaluations of several chemotherapeutics drugs and compared among stemness clusters. As shown in Fig. 3a, the estimated IC50 values of Bosutinib, Docetaxel, Elesclomol, Gefitinib, Lenalidomide, Methotrexate and Sunitinib were significantly lower in Cluster 3, implying that Cluster 3 subtype might be more sensitive to these drugs. Cluster 1 and Cluster 2 were more sensitive to Cytarabine and Lapatinib. Furthermore, we assessed the immunotherapy responses of the three subtypes via TIDE algorithm. Consistent with the TME landscape that Cluster 2 comprised richer infiltrations of tumoricidal cells like CD8 T cells, 327 of 424 patients (77.1%) in Cluster 2 were estimated to benefit from immunotherapy, which was significantly higher than Cluster 1 (271 of 727, 37.3%) and Cluster 3 (92 of 316, 29.1%) (Fig. 3b). Yet increased immunosuppressive M2 macrophages and decreased M1 macrophages as well as CD8 T cells might account for the minimal response rate in Cluster 3.

### WGCNA to identify stemness Cluster 3-accosiated module and hub genes

**Fig. 4:**
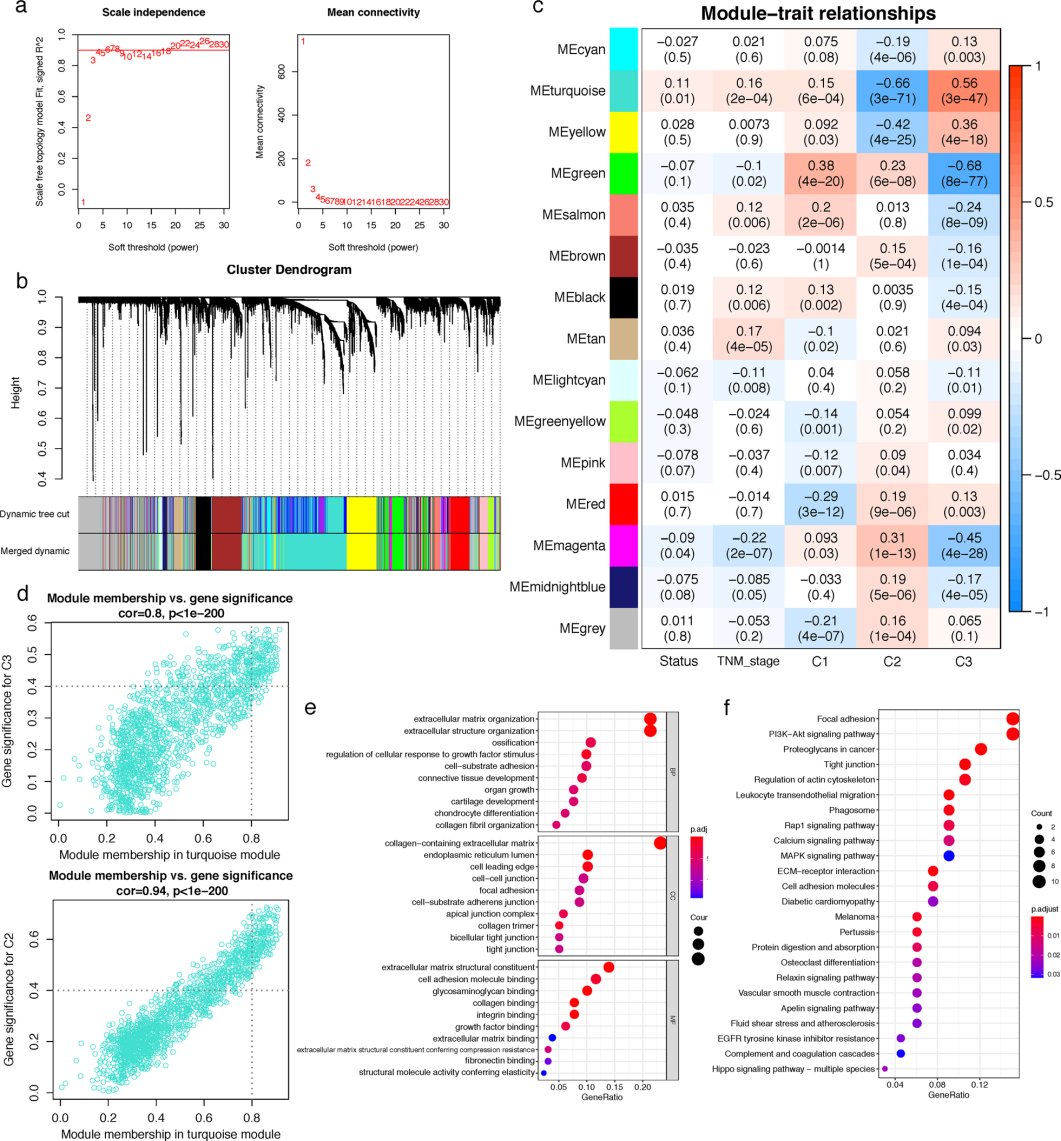
Identification of hub genes by WGCNA for CRC stemness subtypes on GSE39582 dataset. **a** Scale independence and mean connectivity of multiple soft-thresholding powers (*β*) from 1 to 30. **b** The cluster dendrogram developed by the weighted correlation coefficients, genes with similar expression patterns were clustered into co-expression modules, and each color represents a module. **c** Heatmap of the correlation between module eigengenes (MEs) and clinical traits as well as stemness subtypes. **d** Scatter plot displaying relationship of module membership (MM) in turquoise module with gene significance for Cluster C3 (GS). **e** Top ten enriched biological process (BP), cellular component (CC) and molecular function (MF) GO terms of Cluster 3-associated module genes. **f** KEGG analysis of Cluster 3-associated module genes

As Cluster 3 CRC patients experienced the least benefit from immunotherapy and the worst survival, we further performed WGCNA to determine typical genes of this subtype in GSE39582 cohort. Firstly, the optimal soft-threshold power *β* was set as 7 to ensure the scale-free network constructions (scale-free *R*^2^ = 0.9) (Fig. 4a). Then, the least number of genes of each module was set as 30, and the clustering dendrogram manifested that genes with similar expression patterns were clustered into 15 modules (Fig. 4b). Among the 15 modules, the turquoise module revealed the strongest positive correlation with Cluster 3 subtype (ME = 0.56, *P* = 3e−47) as well as the most negative association with Cluster 2 subtype (ME = − 0.66, P = 3e−71) and moderately correlated with survival status (ME = 0.11, *P* = 0.01) and TNM stage (ME = 0.16, *P* = 2e−04) (Fig. 4c). Hence, the turquoise module was chosen as the hub module, from which 138 intersectant candidate hub genes were filtered for further analyses with the filtration criteria of MM > 0.8 and GS > 0.4 (Fig. 4d).

Furthermore, to explore the biological functions of the hub genes in turquoise module, GO and KEGG pathway annotation analyses were performed. The principal enriched GO terms for biological process (BP), cellular component (CC) and molecular function (MF) were extracellular matrix organization and extracellular structure organization, collagen-containing extracellular matrix, extracellular matrix structural constituent. In addition, KEGG analysis revealed the turquoise module was mainly involved in focal adhesion, PI3K-Akt signaling pathway, proteoglycans in cancer, tight junction, regulation of actin cytoskeleton and leukocyte transendothelial migration.

### Construction and validation of prognostic stemness signature based on hub genes of turquoise module

**Fig. 5:**
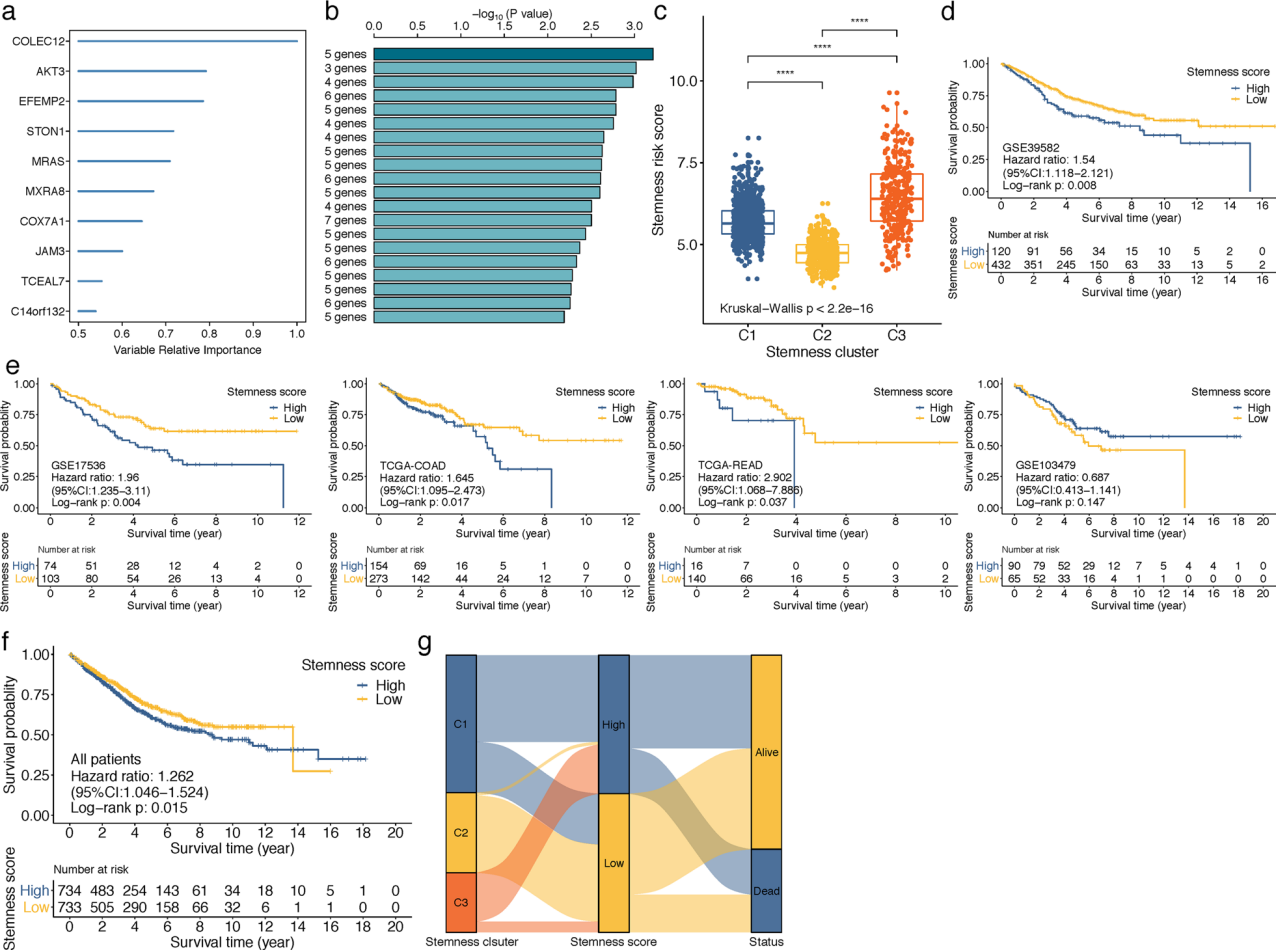
**a** Ten hub genes with relative importance > 0.5 were selected by random survival forest algorithm. **b** Top 20 gene signatures from 1023 (2^10^–1) gene alignment assemblies evaluated by log-rank tests, which were displayed as the −log10 (log‐rank *P*) values, and the 5-gene signature marked in dark blue was chosen as the final model. **c** The distribution of stemness-risk score among different stemness subtypes; ****p < 0.0001. **d** Kaplan–Meier analysis for overall survival difference between high and low stemness-risk score CRC patients in GSE39582. **e** Kaplan–Meier analysis for overall survival difference between high and low stemness-risk score CRC patients in GSE17536, TCGA-COAD, TCGA-READ and GSE103479 cohorts. **f** Kaplan–Meier analyses for overall survival difference between high and low stemness-risk score CRC patients in all CRC patients. **g** Alluvial diagram of distributions for all CRC patients with different stemness clusters, stemness-risk scores and survival outcomes

Firstly, univariate Cox regression analysis was performed on GSE39582 cohort, and we identified 59 genes that were significantly correlated with OS (*P* < 0.05). Then, random forest survival analysis was implemented to further filter out genes with low importance on OS, and 10 genes (COLEC12, AKT3, EFEMP2, STON1, MRAS, MXRA8, COX7A1, JAM3, TCEAL7 and C14orf132) with relative importance > 0.5 were selected (Fig. 5a). Subsequently, we assembled the 10 genes into 1023 (2^10^–1) alignment assemblies, followed by log-rank tests for the prognostic model evaluations. Figure 5b displays the -log_10_ (log‐rank P) values of top 20 ranking models, from which the top-rank signature comprised of five genes (COLEC12, EFEMP2, STON1, TCEAL7 and C14orf132) was extracted for the stemness-risk model construction: Risk score = (0.243 * expression of COLEC12) + (0.183 * expression of EFEMP2) + (0.243 * expression of STON1) + (0.211 * expression of TCEAL7) + (0.297 * expression of C14orf132). Using this formula, the stemness-risk score of each CRC patient was calculated. Notably, stemness C3 had the highest stemness-risk score, while the stemness-risk score was lowest in stemness C2 (Kruskal− Wallis analysis, *P* < 0.0001) (Fig. 5c). Kaplan–Meier analysis showed that patients with high stemness-risk score experienced significantly worse prognosis in comparison with those with low stemness-risk score (log-rank test, P = 0.008) (Fig. 5d). Next, we validated the OS prediction ability of the stemness-risk model on other cohorts, and Kaplan–Meier survival curves showed that except for GSE103479 cohort (log-rank test, *P* = 0.147), high stemness-risk score CRC patients had shorter OS time than low stemness-risk score patients in GSE17536 (log-rank test, *P* = 0.004), TCGA-COAD (log-rank test, *P* = 0.017), TCGA-READ (log-rank test, *P* = 0.037) (Fig. 5e). By choosing the median stemness-risk score as cutoff for all 1,467 CRC patients from the five cohorts, patients in high stemness-risk group also had worse OS than low stemness-risk group patients (log-rank test, *P* = 0.015) (Fig. 5f), and the distribution of these patients in three stemness clusters was displayed in Sankey diagram (Fig. 5g).

### Correlation between stemness-risk signature and TME infiltration patterns

**Fig. 6:**
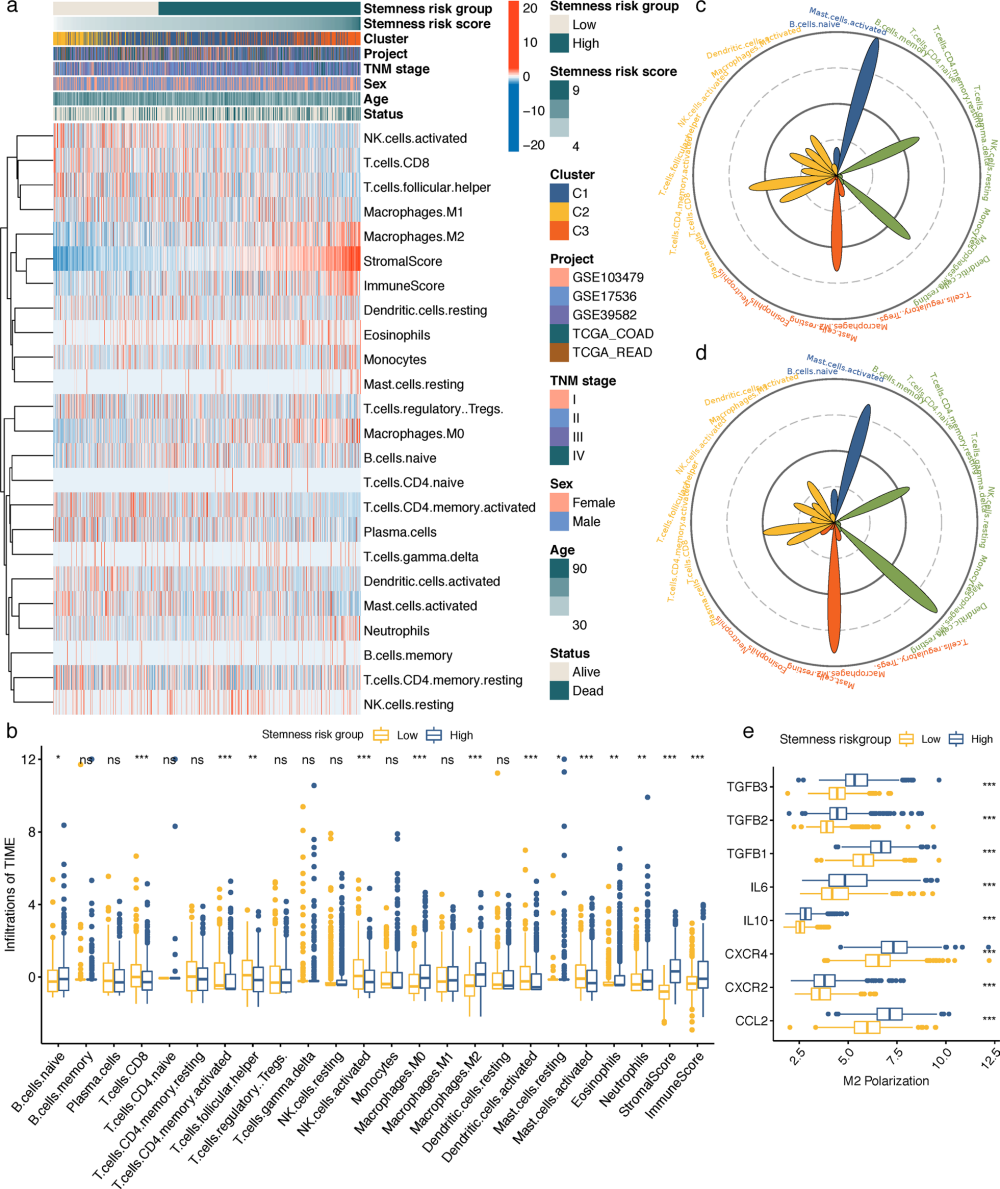
**a** Heatmap manifesting the relationship between TIME infiltration and stemness-risk score as well as clinical pathological parameters. **b** The fraction of TIME cells (z-score transformed) in high and low stemness-risk groups. Wilcoxon test, ns, not significance; *p < 0.05; **p < 0.01; ***p < 0.001. TIME, Tumor immune microenvironment. **c**–**d** Radar charts showing the immune cell infiltration abundances in low (**c**) and high (**d**) stemness-risk groups. **e** Comparison of markers associated with M2 macrophages polarization between high and low stemness-risk groups

After running CIBERSORT and ESTIMATE, 893 samples with CIBERSORT P < 0.05 were obtained, and the proportion of 22 immune cells as well as immune and stromal scores in each CRC sample were calculated and visualized via heatmap (Fig. 6a), from which we observed that with the increase of stemness-risk scores, the fractions of M2 macrophages and M0 macrophages, as well as immune and stromal scores increased, while fractions of activated NK cells, CD8 T cells and T cells follicular helper decreased. Consistently, Wilcoxon analysis revealed that the fractions of B cells naïve, M0 macrophages, M2 macrophages, neutrophils, immune and stromal scores were more abundant in high stemness-risk group. In contrast, several antitumor immune cells were richer in low stemness-risk group, including CD8 T cells, activated memory CD4 T cells, T cells follicular helper, activated NK cells and activated dendritic cells (Fig. 6b), indicating that low stemness-risk group patients might be more sensitive to immunotherapy. The average TME distributions in each group are also displayed in Fig. 6c–d. The low stemness-risk group exhibited an anti-tumor immune immunity, while high stemness-risk group was characterized by higher immunosuppressive cells, and M2 macrophages and M0 macrophages were the most abundant cell types. Meanwhile, we observed M2 polarization regulators were highly expressed in high stemness-risk group (Fig. 6e). Taken together, these results suggest that high stemness-risk group patients might be less sensitive to immunotherapy owing to the stromal and M2 macrophage-mediated immunosuppression effects.

### The value of stemness-risk score in chemotherapy sensitivity and immunotherapy response predictions

**Fig. 7:**
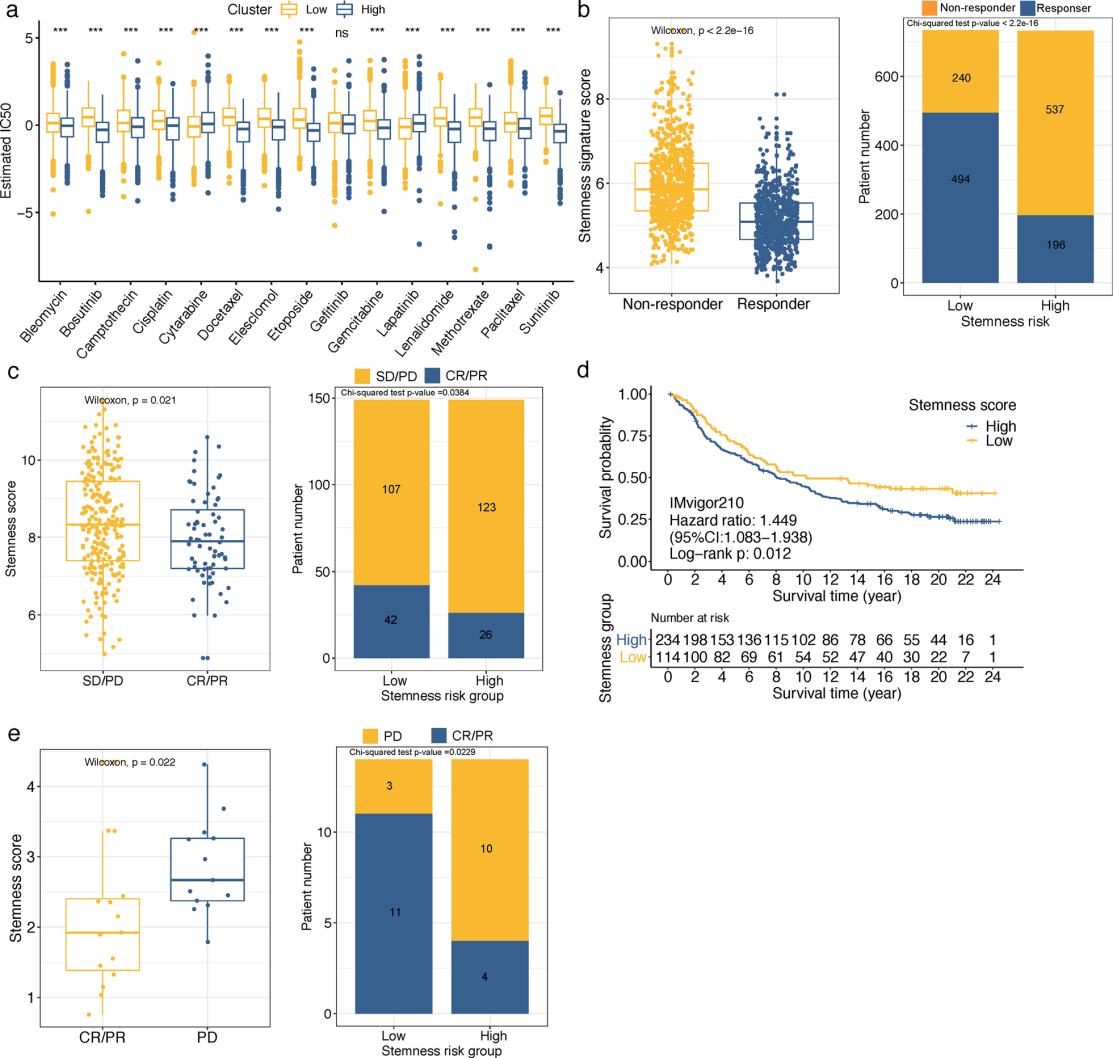
**a** Box plots of the estimated IC50 values of several chemotherapy drugs between high and low stemness-risk groups. Wilcoxon test, ns, not significance; ***p < 0.001. **b** TIDE results of the differences of stemness-risk score in the respond and non-respond groups and distributions of responder and non-responder in distinct stemness-risk groups. **c** Differences of stemness-risk score in SD/PD and CR/PR groups and distributions of anti-PD-L1 therapeutic response in distinct stemness-risk groups in IMvigor210 cohort. **d** Kaplan–Meier survival analysis revealed high stemness-risk score correlated with a worse prognosis in IMvigor210 cohort. **e** Differences of stemness-risk score in PD and CR/PR groups and distributions of anti-PD-1 therapeutic response in distinct stemness-risk groups in GSE78220 cohort

Our aforementioned results have revealed that stemness Cluster C3 was more sensitive to several chemical drugs (Fig. 3a) but less sensitive to immunotherapy response (Fig. 3b). Similarly, we observed that high stemness-risk group was more sensitive to not only these C3-sensitive drugs like Bosutinib, Docetaxel, Elesclomol, Lenalidomide, Methotrexate and Sunitinib, but also Bleomycin, Camptothecin, Cisplatin, Cytarabine, Etoposide, Gemcitabine, Lapatinib and Paclitaxel (Fig. 7a). Subsequently, we analyzed the correlation of the stemness-risk model and immunotherapy response predicted by TIDE method. As shown in Fig. 7b, the stemness-risk score was significantly lower in responder patients than non-responders (Wilcoxon test, *P* < 0.001), and the ratio of immunotherapy responders in the low-risk group was more than two and a half times than that in the high-risk group (67.3 versus 26.74%, Chi-square test, *P* < 0.001).

Moreover, to further test the capability of our model on immunotherapeutic benefit prediction, we applied our model to two real-world immunotherapy cohorts (anti-PD-L1 in IMvigor210 cohort and anti-PD-1 in GSE78220 cohort). As shown in Fig. 7c, in IMvigor210 cohort, patients with complete response (CR) and partial response (PR) to anti-PD-L1 therapy had significantly lower stemness-risk scores than stable and progressive diseases (SD, PD) patients (Wilcoxon test, *P* = 0.021), and patients in the high stemness-risk group underwent significantly less therapeutic benefits than low stemness-risk group (17.45 versus 28.19%, Chi-square test, *P* = 0.0384). In addition, the survival time of high stemness-risk group was significantly shorter than that of the low stemness-risk group (HR = 1.449, 95%CI:1.083–1.983, log-rank *P* = 0.012) (Fig. 7d). Similarly, in GSE78220 cohort, patients who benefited from anti-PD-1 showed lower stemness-risk scores (Wilcoxon test, *P* = 0.022), and the frequency of CR/PR was also significantly higher in low stemness-risk group (78.57 versus 28.57%, Chi-square test, *P* = 0.0229) (Fig. 7e). These findings indicated that CRC patients with low stemness-risk score might be sensitivity to immunotherapy.

### mRNAsi was higher in Cluster 3 and negatively correlated with stemness-risk score

**Fig. 8:**
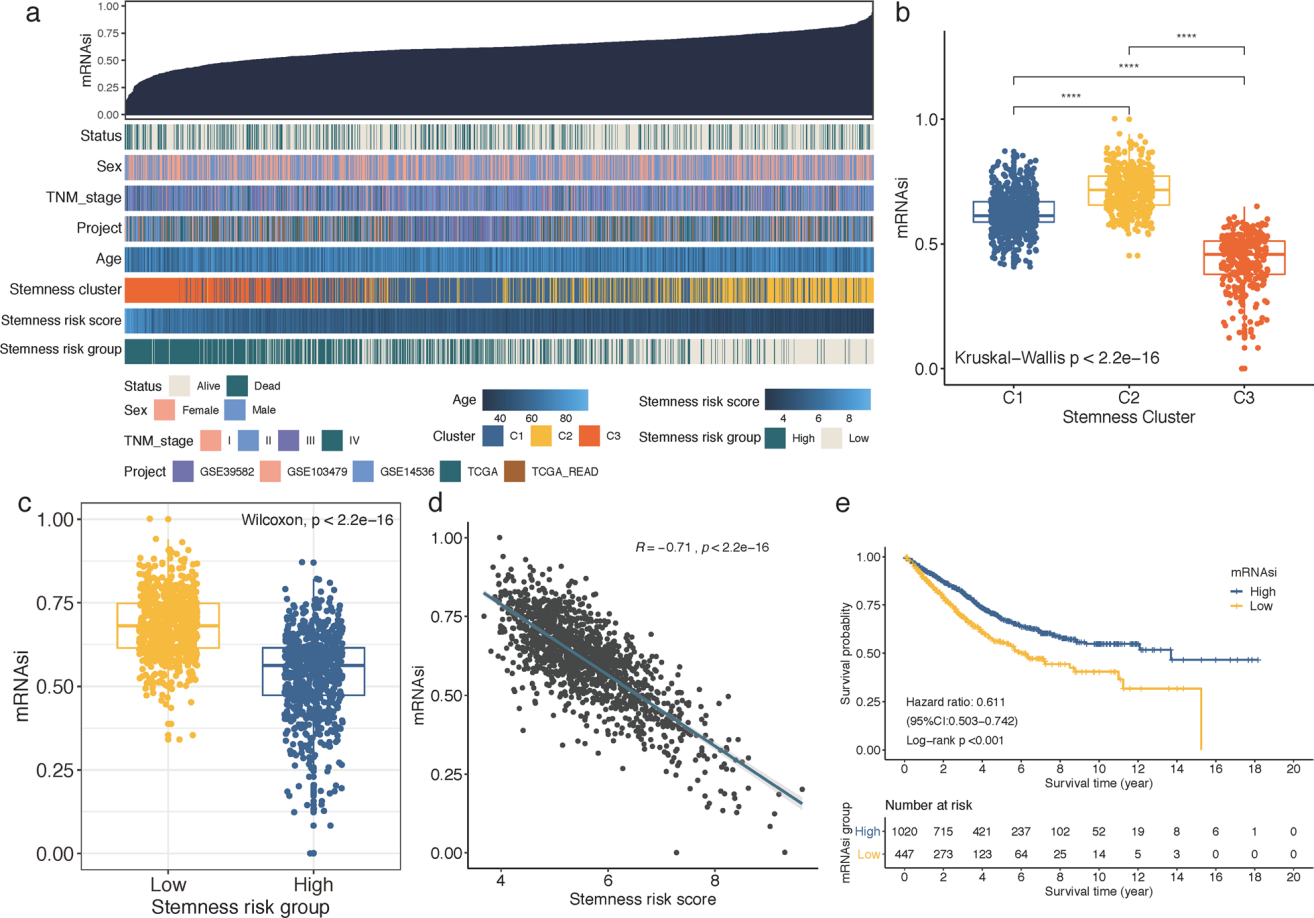
**a** The overview of correlation between mRNAsi and clinical features as well as stemness clusters and risk scores. **b**–**c** Box plot of the comparison of the mRNAsi between three stemness Clusters (**b**) and stemness-risk groups (**c**); ****p < 0.0001. **d** The Spearman correlation of stemness-risk score with mRNAsi. **e** Kaplan–Meier survival analysis revealed high mRNAsi correlated with a better prognosis in CRC patients

By applying OCLR algorithm, mRNAsi of each CRC patients was calculated based on the gene expression profiles, and we then examined the correlations between mRNAsi and the stemness subtypes. As displayed in Fig. 8a, by ranking mRNAsi from low (left) to high (right), we observed that stemness Cluster 3 was mainly concentrated in low mRNAsi regions, while stemness Cluster 2 had the highest mRNAsi, which was confirmed by the comparison analysis (Fig. 8b). Similarly, mRNAsi was significantly higher in low stemness-risk group (Wilcoxon test, *P* < 0.001) (Fig. 8c), and mRNAsi was highly and negatively correlated with the stemness-risk score (Spearman correlation = − 0.71, *P* < 0.001) (Fig. 8d). Furthermore, Kaplan–Meier analysis indicated that CRC patients with low-mRNAsi experienced longer OS than high-mRNAsi patients (Fig. 8e).

### GSEA analysis for differences of hallmark gene sets between high and low stemness-risk groups

**Fig. 9:**
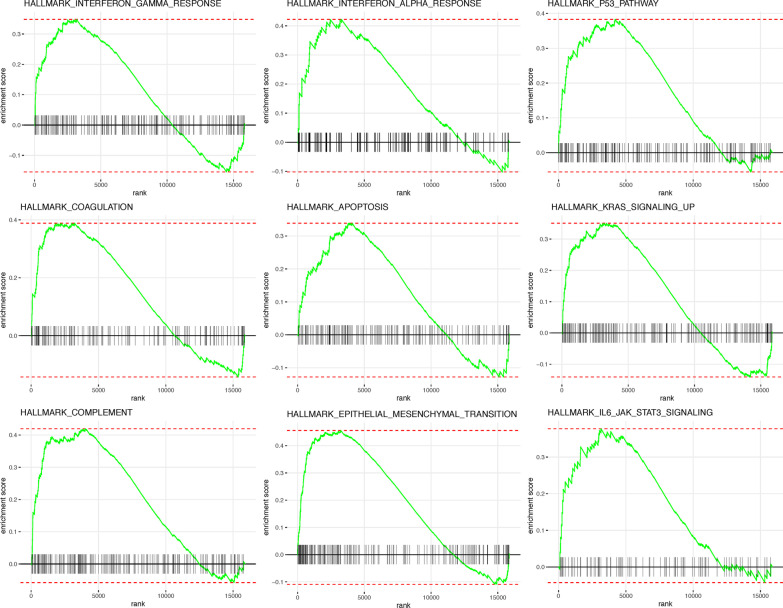
Gene set enrichment analysis (GSEA) of hallmark gene sets between high and low stemness-risk groups

GSEA was performed to investigate the differentially enriched hallmark gene sets between high and low stemness-risk groups. As shown in Fig. 9, genes highly expressed in high stemness-risk group were significantly enriched in several hallmark gene sets, including interferon gamma response, interferon alpha response, P53 pathway, coagulation, apoptosis, KRAS signaling upregulation, complement, epithelial–mesenchymal transition (EMT) and IL6-mediated JAK-STAT signaling gene sets.

### Knockdown of COLEC12 attenuates CSC traits of CRC cells

**Fig. 10:**
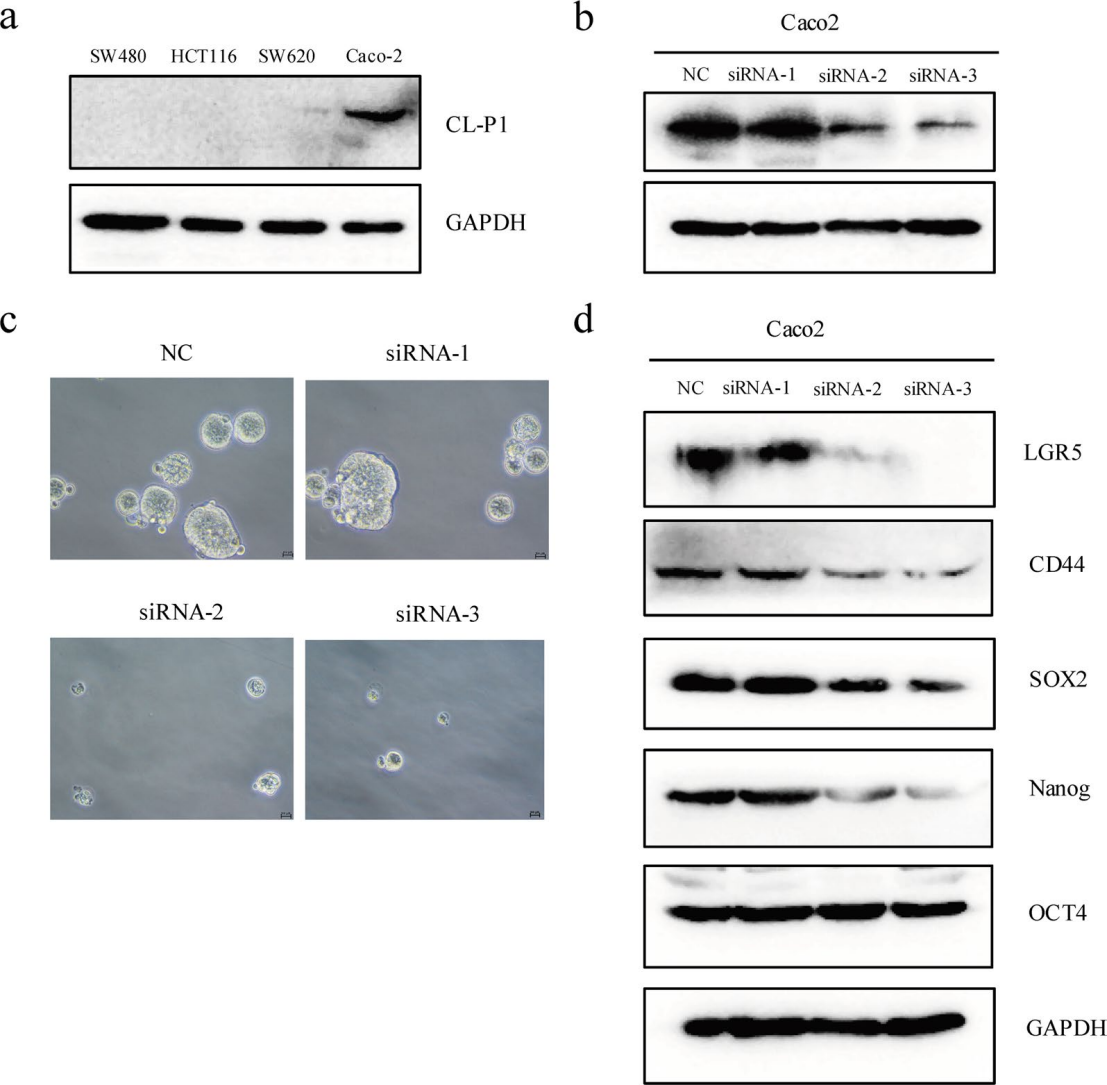
**a** Western blot analysis of COLEC12 protein levels in four kinds of CRC cell lines. **b** COLEC12 protein expression was successful inhibited by specific siRNA against COLEC12 (si- COLEC12-2 and -3) compared with negative control (NC). **c** Representative images of Caco-2 cell spheres after transfection with NC and si-COLEC12-1, -2, and -3 for 5 days, respectively. **d** Western blot showed the expression of stem-related markers LGR5, CD44, SOX2 and NANOG was down-regulated in si- COLEC12-2 and -3 transfected Caco-2 cells

Among all the 5 genes in the signature, COLEC12 was identified as the most significant one via random forest survival analysis, and we therefore functionally verified the potential role of COLEC12 in promoting CSC properties. As shown in Fig. 10a, western blot assay revealed the protein level of COLEC12 was significantly higher in Caco-2 cell line than that in others, and specific siRNAs were then utilized to successfully inhibit COLEC12 expression in Caco-2 cells (si-COLEC12-2 and si-COLEC12-3; Fig. 10b). Following, sphere formation assay was performed to assess the stemness traits of COLEC12, and the results indicated that the sphere numbers and sizes were markedly attenuated after si-COLEC12-2 and -3 transfections in Caco-2 cells and suggested a suppression of CRC cells that exhibit stemness properties (Fig. 10c). In addition, we examined the protein expressions of stem cell markers LGR5, CD44, SOX2, NANOG and OCT4 by western blot, and results indicated the LGR5, CD44, SOX2 and NANOG protein levels were decreased after si-COLEC12-2 and -3 transfections (Fig. 10d), indicating that COLEC12 affected the CSC traits of CRC cells.

### Comparison of the stemness-risk score with other CRC stemness models

**Fig. 11:**
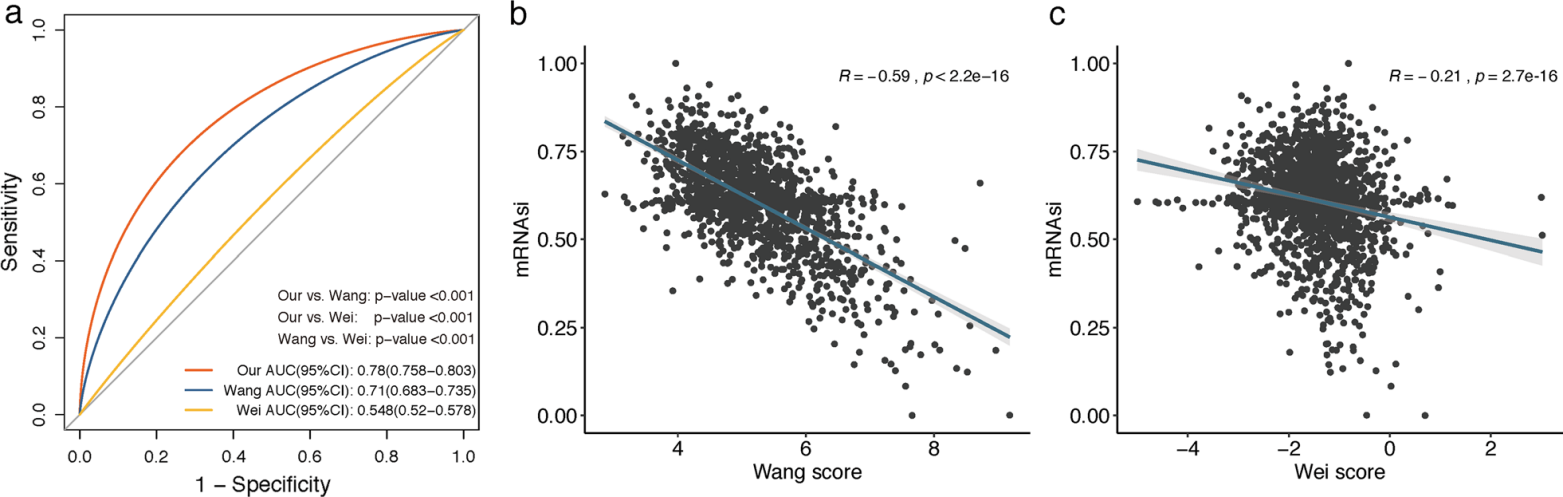
**a** The ROC curves of our 5 gene, Wang-15 gene and Wei-3 gene signatures to predict immunotherapy response in the entire CRC cohort. **b** The Spearman correlation of Wang-15 gene signature with mRNAsi. **c** The Spearman correlation of Wei-3 gene signature with mRNAsi

Based on the stemness index (mRNAsi), Wang et al. [48] and Wei et al. [49] both performed WGCNA and, respectively, identified 15-mRNA- and 3-mRNA mRNAsi-related signature. To determine whether our stemness subtype-related five-gene signature is superior to these models, we firstly compared the predictive capability of the three models in predicting immunotherapy response estimated via TIDE analysis on the entire 1467 CRC patients. The receiver operating characteristic (ROC) curve revealed the predictive performance of our 5-gene stemness model (area under the curve (AUC) (95%CI): 0.78 (0.758–0.803)) was significantly better than models from Wang (AUC (95%CI): 0.71 (0.683–0.735)) and Wei (AUC (95%CI): 0.548 (0.52–0.578)) (both *P* < 0.001) (Fig. 11a). Subsequently, we performed Spearman correlation analysis between each model and mRNAsi, and as noted earlier in this paper, our model was highly and negatively correlated with the mRNAsi (Spearman correlation = − 0.71, *P* < 0.001) (Fig. 8d), while the correlation coefficient was moderate in Wang’s model (Spearman correlation = − 0.59, *P* < 0.001) (Fig. 11b) and weak in Wei’s model (Spearman correlation = − 0.21, *P* < 0.001) (Fig. 11c), which further indicated our model could serve as a robust predictor in reflecting stemness of CRC samples.

## Discussion

CSCs are highly heterogeneous cell populations that exist in dynamic equilibrium with their intricate intrinsic and ambient microenvironments [50]. Phenotypic characterization of CSC properties would contribute to the design of molecular agents targeting both CSCs and unfavorable TME [51]. Here, this study presents the first systematic bioinformatic analyses to uncover the molecular characteristics of 26 CSCs gene sets on large-scale cohorts of multicenter CRC patients. We suggest that precise molecular subtyping of stemness characteristics would prospectively stratify CRC patients with different prognosis, TME infiltration patterns and therapeutic responses; characterization of CSCs subtype-related gene expression pattern could be novel strategy for the guidance of more efficient patient-specific therapy.

Among the three diverse stemness subtypes identified via unsupervised clustering based on ssGSEA scores of 26 stemness gene sets, C2 subtype was characterized by higher enrichment levels in CSCs gene sets with favorable prognosis, richer infiltrations of antitumor TME patterns like CD8+ T cells, activated memory CD4+ T cells, T cells follicular helper and activated NK cells, and greater sensitivity to immunotherapies. Conversely, the stemness cluster 3 enriched highly in CSCs gene sets with adverse prognosis and presented an immunosuppressive phenotype with higher infiltrations of M2 macrophages and stromal scores and thus had the lowest sensitivity to immunotherapies. To further elucidate the genomics characteristics of the stemness subtypes, we performed the in-depth WGCNA analysis for the recognition of co-expressed, stemness cluster-related gene modules, and the turquoise module, which was most positively relevant to stemness C3 but negatively correlated with stemness C2, was identified as the most crucial module for further analysis. GO analysis revealed top biological processes of turquoise module were extracellular matrix organization, and extracellular structure organization, collagen-containing extracellular matrix and extracellular matrix structural constituent were main terms for cellular components and molecular functions. Studies have reported that mild changes in ECM composition would aggravate the invasive features of tumor cells through mechano-transduction [52, 53] and facilitate stemness and metastasis [54, 55]. Then, univariate Cox and random forest survival analyses were performed for the mining of prognostic hub genes in turquoise module, and through the 1023 combinations of ten hub genes, a stemness prognostic signature comprising five genes (COLEC12, EFEMP2, STON1, TCEAL7 and C14orf132) was constructed to quantify the stemness pattern. Consistently, stemness C3 had the highest stemness-risk score, and higher stemness-risk score correlated with worse prognosis for CRC patients.

In terms of TME patterns, advances in the recognition of interactions between CSCs and TME could provide novel insights for the understanding of CSC-mediated immunomodulation and design of CSC-based therapies [56, 57]. Currently, chimeric antigen receptor (CAR)-T-cell-based immunotherapies targeting CSCs have achieved promising efficacy for hematological pathologies but not for solid tumors like CRC [58]. Solid cellular barrier, immunosuppressive microenvironment and lack of tumor-specific antigens have hindered the clinical application of CAR-T in solid tumors [58]. Schofield firstly proposed the existence of stem cell niches, as represented by specific microenvironments, that are essential for the self-renew and differentiate of CSCs [59], and this TME niche recruited immunosuppressive cells like cancer-associated fibroblasts, Tregs and M2 macrophages to augment their pro-tumorigenic activities [60]. Along this line, accumulating evidence revealed that both cell-intrinsic modifications and microenvironmental perturbations propelled the reversible and dynamical shift of CSCs between stem-like and differentiated status [61]. Thus, molecular features with respect to CRC stemness remain uncovered, and a common and reliable signature reflecting stemness feature is still lacking. Simultaneously, additional investigations to interfere with stemness, enhance immune cell penetration and affect the suppressive TME components would make therapies like CAR-T more promising in solid tumors, reaching the levels observed in blood cancers. In our analysis, we found that low stemness-risk CRC patients were characterized by antitumor immunity with high infiltrations of NK cells, CD8 T cells and T cells follicular helper, while the immunosuppressive M2 macrophages and stromal components were richer in high stemness-risk group. We also found that M0 macrophages and M2 macrophages were the most plentiful cell types in high stemness-risk group, suggesting that M2-oriented polarizations might play main roles in mediating immunosuppression and enhancing aggressiveness of the tumor [62, 63]. Notably, we observed elevated levels of M2 polarity factors (TGF-β, IL-6 and IL-10) in high stemness-risk group. Recent studies showed that CSCs promoted the polarization of M2 macrophages and the recruitment of tumor-associated neutrophils (TANs), myeloid-derived suppressor cells (MDSCs) by inducing the abnormal expression of MHC-I molecules and the secretion of immunosuppressive cytokines, which were conducive to the formation of tumor immunosuppressive microenvironment [64, 65]. Tumor-associated macrophages (TAMs) are the principal source of these pro-inflammatory cytokines, which in turn drive macrophages into M2-polarized subtype [66] that interactively contribute to the EMT and CSCs transition [67–69] as well as immunosuppression and therapy resistance [70]. With respect to immunotherapy guidance, TIDE analysis revealed that a lower stemness-risk score correlated with a higher immunotherapy response for CRC patients. When applying our model to the IMvigor210 and GSE78220 trials, synonymous results were obtained that low stemness-risk patients received better clinical benefits following anti-PD-L1 and anti-PD-1 therapies, which affirmed the predictive validity of our stemness model.

Based on dataset of pluripotent stem cell and their progenitors [71, 72], Malta et al. designed transcriptional stem cell index (mRNAsi) via OCLR method to quantitatively evaluate CSCs activities and malignant cells dedifferentiation of around 12,000 tumor samples in TCGA [45]. As TCGA solid tumors presented distinct levels of stemness features [45], our findings were consistent with previous studies that CRC patients with higher mRNAsi indices experienced better prognosis [48, 73], although the general recognitions of CSCs are their mediations of tumorigenesis, metastasis and recurrence. However, considerable heterogeneity exists among CSCs with regard to markers and models that describe cancers [74]. Alternatively, we identified three stemness clusters based on multi CSCs gene sets and designed stemness-risk model, which displayed a strong negative correlation with mRNAsi (cor = -0.71). In GSEA, besides the enrichment in immune-related hallmarks, high stemness-risk group was also enriched in P53 pathway, coagulation, apoptosis, KRAS signaling and EMT. EMT has been regarded as the initiator that underlies the acquisition of malignant features by carcinoma cells [75], mounting evidence proves the trigger of EMT is closely associated with the acquisition of CSCs traits by both normal and neoplastic cells [76, 77], and non-CSCs are also generated with many CSCs features during the EMT evolvements [78, 79]. To sum up, we speculate our model was capable of reflecting the stemness characteristics in CRC.

For the five stemness model genes identified in this study, COLEC12 has been suggested as a prospective biomarker for anaplastic thyroid cancer [80], and Li et al. reported that knockdown of COLEC12 could promote apoptosis and enhance inflammation through TLR4 in osteosarcoma [81]. EFEMP2 has been regarded as an auspicious biomarker for CRC early detection [82], and knockdown of EFEMP2 could inhibit the proliferation and invasion of CRC cells via ERK1/2 pathway [83]. Huang et al. reported malignant glioma samples with higher EFEMP2 expressions were more prone to exhibit M0 macrophages features [84]. High expressions of STON1 and C14orf132 were correlated with worse prognosis in bladder urothelial carcinoma [85] and CRC [86], respectively. Mao et al. performed WGCNA and identified TCEAL7 as hub gene correlated with unfavorable prognosis and stemness features in gastric cancer [87], while TCEAL7 has been regarded as a tumor suppressor in glioblastoma [88] and ovarian cancer [89].

Inevitably, several limitations need to be illustrated in this study. First, the data in our research were acquired from the public databases rather than our database; although there were sufficient CRC samples as the verification set to support the conclusions of our research, we need to further validate the prognostic and therapeutics effects of this model with large sample size from our own center in the future. Second, the model genes associated with CRC stemness were identified based on bioinformatics, and further functional experiments are necessitated for the investigation of their biological mechanisms on stemness and TME landscape, as well as their capabilities as targets to improve immunotherapy and chemotherapy efficacy. Third, since public transcriptomic data of CRC patients receiving immunotherapy are currently very limited, the actual relationship between stemness subtypes as well as stemness-risk groups and immunotherapy responsiveness in CRC needs to be evaluated in an immunotherapy cohort in the future.

## Conclusion

Taken together, through unsupervised cluster on stem cell gene sets, three stemness-associated subtypes with diverse prognosis, TME patterns and therapy responses were systematically identified for the first time. By applying WGCNA, Cox and random survival forest analyses, a five-gene stemness-associated risk model was constructed and validated in large cohorts of CRC patients. We suggest our stemness model has prospective clinical implications for prognosis evaluation and might facilitate physicians selecting prospective responders for preferential use of current immune checkpoint inhibitors.

## Supplementary Information

**Additional file 1. Fig. S1**. Spearman’s correlation analyses of the 26 stemness ssGSEA scores in 1,467 CRC samples.

**Additional file 2. Table S1**. The twenty-six stem gene sets acquired from StemChecker database to identify colorectal cancer subtype.

## Data Availability

The datasets supporting the findings of this study are available in the from The Cancer Genome Atlas (TCGA) (https://gdc.xenahubs.net) and GEO (https://www.ncbi.nlm.nih.gov/geo/) databases. Further inquiries can be directed to the corresponding author.

## Abbreviations

CRC: Colorectal cancer
CSCs: Cancer stem cells
TME: Tumor microenvironment
ssGSEA: Single-sample gene set enrichment analysis
WGCNA: Weighted gene correlation network analysis
TCGA: The Cancer Genome Atlas
GEO: Gene Expression Omnibus
FPKM: Fragments per kilobase per million mapped reads
TPM: Transcripts per million
OS: Overall survival
TF: Transcription factor
GDSC: Genomics of Drug Sensitivity in Cancer
TIDE: Tumor Immune Dysfunction and Exclusion
MAD: Median absolute deviation
TOM: Topological overlap matrix
ME: Module eigengene
GS: Gene significance
MM: Module membership
GO: Gene Ontology
KEGG: Kyoto Encyclopedia of Genes and Genomes
RFS: Random survival forest
mRNAsi: Messenger RNA expression-based stemness index
OCLR: One-class logistic regression
GSEA: Gene set enrichment analysis
EMT: Epithelial–mesenchymal transition
DMEM: Dulbecco’s modified Eagle’s medium
FBS: Fetal bovine serum
siRNA: Small interfering RNA
NC: Negative control
WB: Western blotting
COLEC12: Collectin subfamily member 12
SOX2: SRY-box transcription factor 2
OCT4: Organic cation/carnitine transporter4
GAPDH: Glyceraldehyde-3-phosphate dehydrogenase
BSA: Bovine serum albumin
AKT3: AKT serine/threonine kinase 3
EFEMP2: EGF-containing fibulin extracellular matrix protein 2
STON1: Stonin 1
MRAS: Muscle RAS oncogene homolog
MXRA8: Matrix remodeling-associated 8
COX7A1: Cytochrome c oxidase subunit 7A1
JAM3: Junctional adhesion molecule 3
TCEAL7: Transcription elongation factor A like 7
C14orf132: Chromosome 14 open reading frame 132
TAN: Tumor-associated neutrophils
MDSCs: Myeloid-derived suppressor cells
CAR: Chimeric antigen receptor
