# Supplementary figures and images for: Characterization of stem cell landscape and identification of stemness-relevant prognostic gene signature to aid immunotherapy in colorectal cancer
Source: Stem Cell Res Ther. 2022 Jun 9;13:244. doi: 10.1186/s13287-022-02913-0 (PMC9185878; doi:10.1186/s13287-022-02913-0)

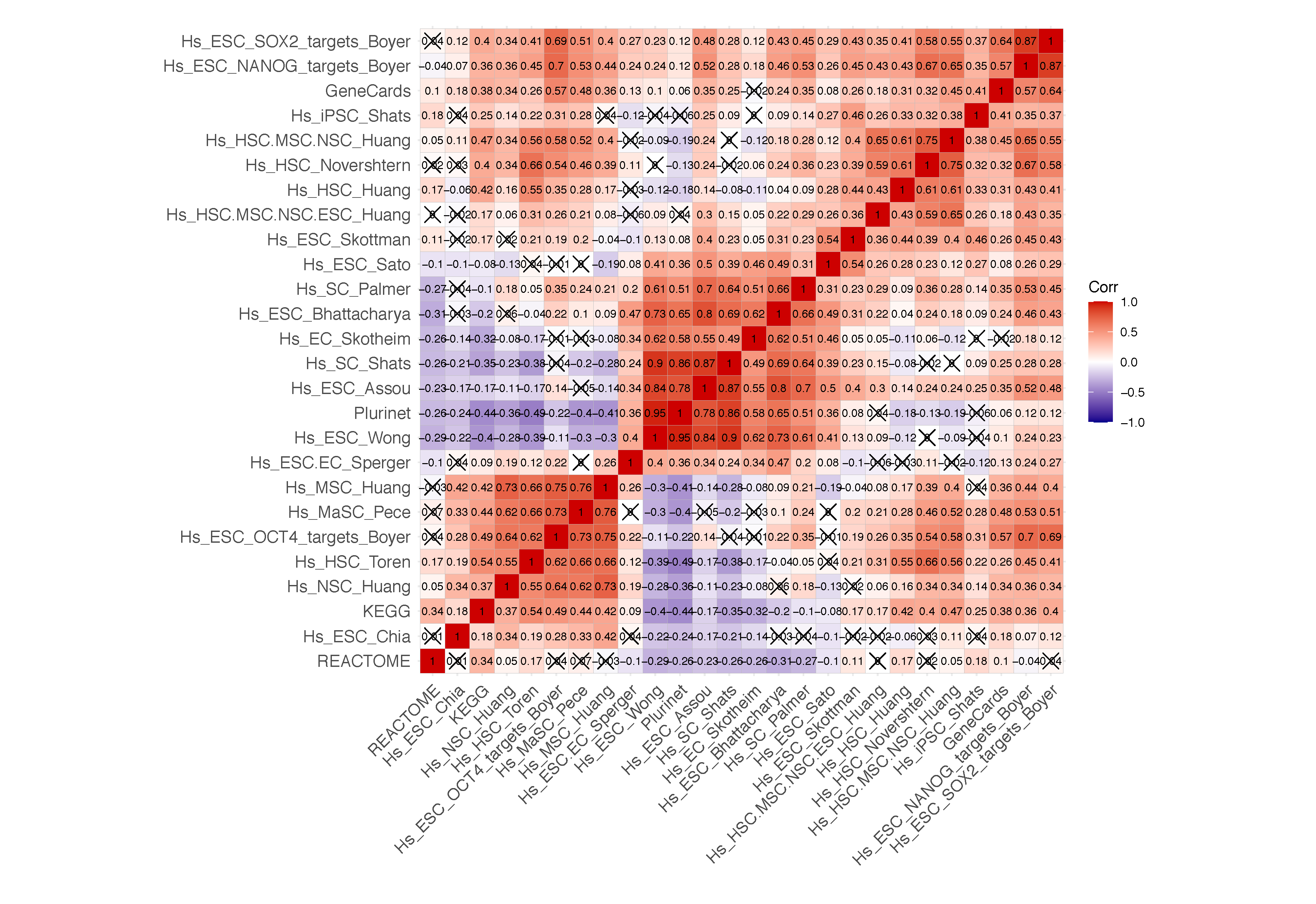

Supplement: Supplementary file 1 — Additional file 1. Fig. S1. Spearman’s correlation analyses of the 26 stemness ssGSEA scores in 1,467 CRC samples. [file 13287_2022_2913_MOESM1_ESM.tiff]
